# Anti-Pigmentary Effect of (-)-4-Hydroxysattabacin from the Marine-Derived Bacterium *Bacillus* sp.

**DOI:** 10.3390/md15050138

**Published:** 2017-05-13

**Authors:** Kyuri Kim, Alain S. Leutou, Haein Jeong, Dayoung Kim, Chi Nam Seong, Sang-Jip Nam, Kyung-Min Lim

**Affiliations:** 1 College of Pharmacy, Ewha Womans University, Seoul 03760, Korea; kyuri0912@naver.com; 2 Department of Chemistry and Nano Science, Global Top 5 Program, Ewha Womans University, Seoul 03760, Korea; leutoualain@yahoo.fr (A.S.L.); h151577@naver.com (H.J.); kdya0503@gmail.com (D.K.); 3 Department of Biology, College of Life Science and Natural Resource, Sunchon National University, Suncheon 57922, Korea; scnu@sunchon.ac.kr

**Keywords:** *Bacillus* sp., (-)-4-hydroxysattabacin, melanogenesis, marine-derived, anti-pigmentary effect

## Abstract

Bioactivity-guided isolation of a crude extract from a culture broth of *Bacillus* sp. has led to the isolation of (-)-4-hydroxysattabacin (**1**). The inhibitory effect of (-)-4-hydroxysattabacin (**1**) was investigated on melanogenesis in the murine melanoma cell line, B16F10, and human melanoma cell line, MNT-1, as well as a pigmented 3D-human skin model. (-)-4-Hydroxysattabacin treatment decreased melanin contents in a dose-dependent manner in α-melanocyte stimulating hormone (α-MSH)-stimulated B16F10 cells. Quantitative real time PCR (qRT–PCR) demonstrated that treatment with (-)-4-hydroxysattabacin down-regulated several melanogenic genes, including tyrosinase, tyrosinase-related protein 1 (TRP-1), and tyrosinase-related protein 2 (TRP-2) while their enzymatic activities were unaffected. The anti-melanogenic effects of (-)-4-hydroxysattabacin were further demonstrated in a pigmented 3D human epidermal skin model, Melanoderm^TM^, and manifested as whitening and regression of melanocyte activation in the tissue.

## 1. Introduction

The skin plays a vital role as a physiological barrier for the inner body against harmful factors. Keratinocytes and melanocytes constitute the epidermis, the outermost layer of the skin, wherein melanin, a key molecule for the defense against harmful UV irradiation and reactive oxygen species (ROS), is synthesized [1]. Specifically, melanin synthesis takes place in melanosomes, membrane-bound organelles in melanocytes that are transferred to adjacent keratinocytes [2]. Melanin determines the color of hair, skin, and eyes, depending on its quantity, quality, and distribution [3]. Accordingly, disorders of melanin synthesis may often lead to hyperpigmentation of the skin, in which several melanogenic factors are critical [4,5].

A number of compounds, such as hydroquinone, arbutin, and kojic acids have been employed as hypopigmenting agents but skin irritation, contact dermatitis, or ochronosis may occur following the chronic exposure to these compounds. Against this backdrop, the search for new, active hypopigmenting agents with improved safety and efficacy has continued. Nature is a major source for this effort and previous studies have identified possible anti-pigmentary effects of molecules derived from natural compounds [6]. However, anti-pigmentary compounds derived from marine microbes are relatively uncommon.

Tyrosinase, tyrosinase-related proteins 1 (TRP-1), and tyrosinase-related proteins 2 (TRP-2) are crucial enzymes for melanogenesis. Tyrosinase (monophenol or o-diphenol, oxygen oxidoreductase, EC 1.14.18.1, syn. polyphenol oxidase) is essential for initiating melanogenesis in melanocyte by oxidizing l-tyrosine to dopaquinone (DO) and the resulting quinone will serve as a substrate for the synthesis of eumelanin and pheomelanin [7,8,9]. Thus, inhibition of catalytic action of tyrosinase is major motivation for attenuating melanogenesis in many cosmetics or pharmaceutical fields [8].

TRP-1 and TRP-2 share about forty percent amino acid homology with tyrosinase [10], and are known to stabilize and increase the activity of tyrosinase. α-Melanocyte stimulating hormone (α-MSH), a potent stimulator of melanin formation in most cultured murine melanoma cell lines and some human melanoma lines, acts through the transcriptional regulation of these melanogenic enzymes as do other pro-melanogenic stimuli such as UV and adrenocorticotropin (ACTH) [11,12]. Accordingly, regulation of these enzymes may be an important strategy for preventing cutaneous pigmentation [13,14,15]. 

During a search for new hypopigmenting agents from marine microbial natural products, a crude extract of culture broth from *Bacillus* sp. (SCO-147) was found in Gwangyang Bay in South Korea, and displayed anti-melanogenic activity. Bioactivity-guided isolation of this extract has led to isolation of (-)-4-hydroxysattabacin (**1**, 4OH-ST) and (-)-sattabacin (**2**). 4OH-ST and sattabacin were first isolated from a *Bacillus* sp. and were found to exhibit antiviral activities against herpes simplex virus types 1 (HSV1) and 2 (HSV2) [16,17]. The biological activity of these natural products on anti-melanogenic effects, in particular, (-)-4OH-ST and (-)-sattabacin has not been reported. We discovered the efficacy of (-)-4OH-ST (**1**) as an anti-melanogenic agent. The mechanism underlying the anti-melanogenic effects of (-)-4OH-ST (**1**) was elucidated along with confirmation of its activity in a 3D pigmented human epidermis model to propose it as a new natural hypopigmenting agent.

## 2. Results and Discussion

### 2.1. Identification of (-)-4-Hydroxysattabacin and (-)-Sattabacin

The ^1^H nuclear magnetic resonance (NMR) spectrum of **1** displayed ortho-coupled aromatic protons [δ_H_ 7.09 (2H, d, *J* = 8.1 Hz), 6.70 (2H, d, *J* = 8.1 Hz)], down-fielded methine protons [4.37 (1H, dd, *J* = 7.5, 4.4 Hz), 3.08 (1H, dd, *J* = 14.3, 4.4 Hz)], and methyl doublets [0.94 (6H, d, *J* = 6.7 Hz)]. The ^13^C NMR spectrum of **1** showed thirteen carbon signals including one carbonyl (δ_C_ 211.6), six aromatics [δ_C_ 154.9, 130.6, 128.5, 115.6], and one oxygenated carbons (δ_C_ 77.8). Compound **1** was identified as 4-hydroxysattabacin based on the comparison of NMR spectroscopic data of **1** to those of a previously reported one [16]. However, a negative value of the optical rotation for **1** suggested that **1** possessed the *R* configuration for C-2′ and was (-)-4-hydroxysattabacin [18]. Comparison of NMR spectroscopic data of **2** to those of the reported one also permitted that the planar structure of **2** was identified as sattabacin [16]. However, the negative sign of the optical rotation indicated that **2** was (-)-sattabacin. The (-)-sattabacin was first isolated from the thermophilic bacterium *Thermosporothrix hazakensis* SK20-1^T^ [19].

### 2.2. Effect of (-)-4-Hydroxysattabacin on the Melanin Synthesis and Cell Viability of B16F10 Cell

**Figure 1:**
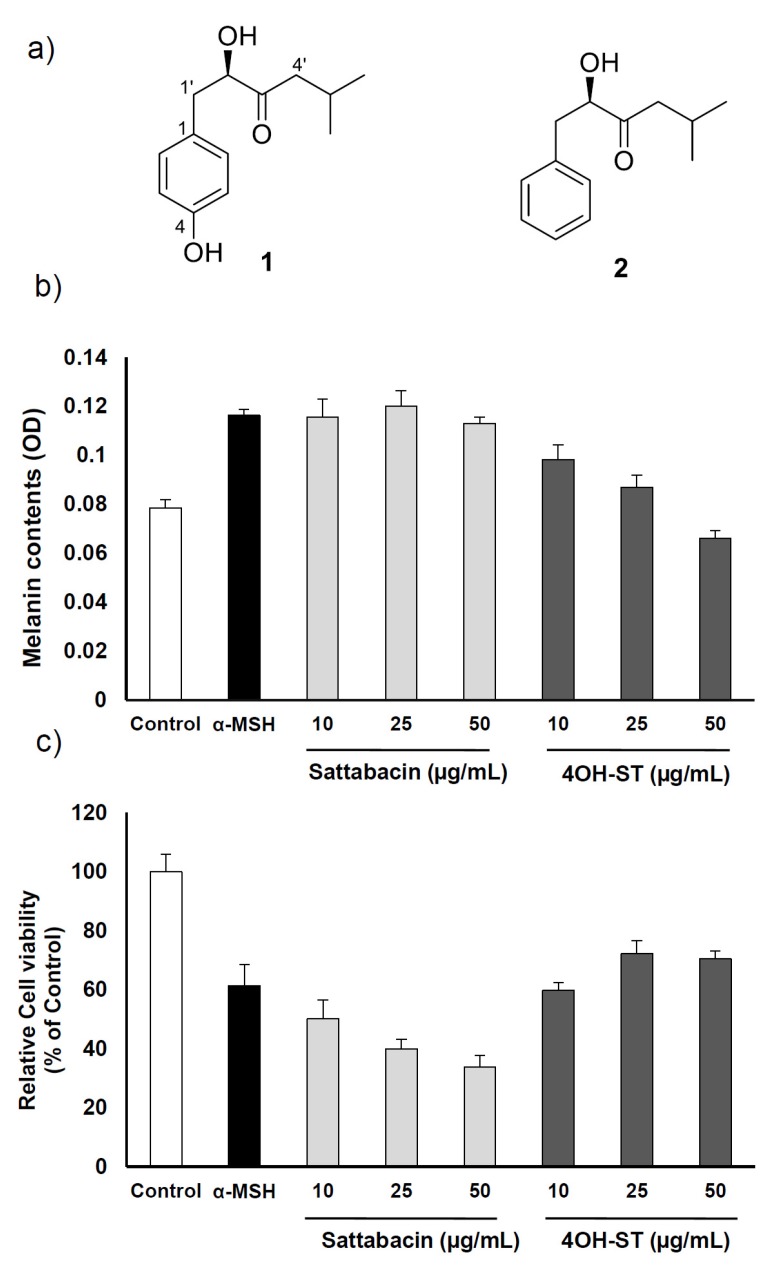
Effects of (-)-4-hydroxysattabacin (4OH-ST, **1**) and (-)-sattabacin (**2**) on melanin contents and cell viability on B16F10 cells. (**a**) Chemical structures of (-)-4OH-ST (**1**) and (-)-sattabacin (**2**); (**b**) Melanin contents were measured by melanin assay; (**c**) Cell viability was determined by WST-1 assay. The cells were treated with arbutin at 50 µg/mL, (-)-4OH-ST (**1**) or (-)-sattabacin (**2**) at the indicated concentrations for 72 h. Data are presented as the mean ± standard deviation (SD) (*n* = 3–5).

**Figure 2:**
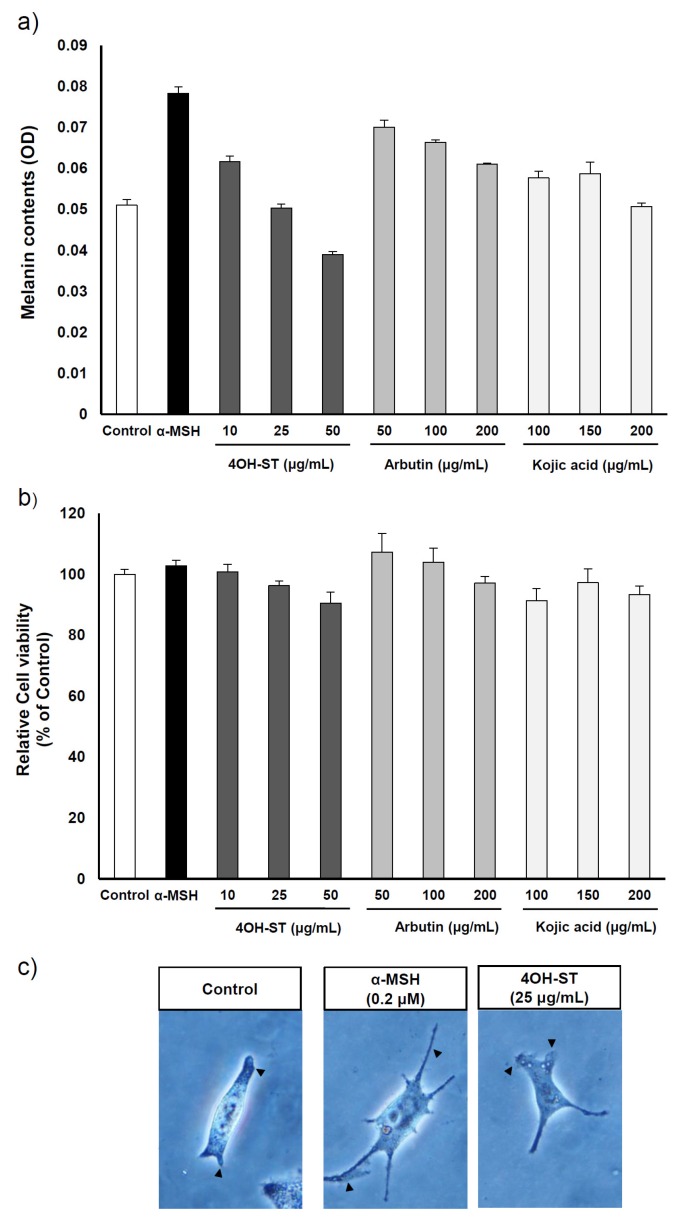
Effects of (-)-4-hydroxysattabacin (4OH-ST), **1**) treatment on B16F10 cells with various concentrations of conventional agents. (**a**) Melanin contents were measured by melanin assay; (**b**) Cell viability was determined by MTT assay. The cells were treated with (-)-4OH-ST (**1**), arbutin, or kojic acid at the indicated concentrations for 48 h; (**c**) Morphological changes of B16F10 cells were observed under optical microscopy (400×). Data are presented as the mean ± SD (*n* = 3).

The effects of (-)-4OH-ST (**1**) and (-)-sattabacin (**2**) (Figure 1a) on melanogenesis were assessed on the α-MSH-stimulated murine melanoma cells, B16F10. 4OH-ST (**1**) showed potent effects on melanogenesis in a dose-dependent manner with minimal effects on cell viability. However, (-)-sattabacin (**2**) was cytotoxic to α-MSH-stimulated murine melanoma cells (Figure 1b,c) and was excluded from further evaluation. Potency and efficacy of the anti-melanogenic activities of **1** were comparable or superior to those of arbutin or kojic acid, well-known hypopigmenting agents (Figure 2a,b). The anti-melanogenic activities of **1** were further confirmed by a visual analysis showing that dendrites extended after α-MSH application were significantly regressed by (-)-4OH-ST application (Figure 2c).

### 2.3. Mechanism Underlying the Anti-Melanogenic Activities of (-)-4OH-ST

**Figure 3:**
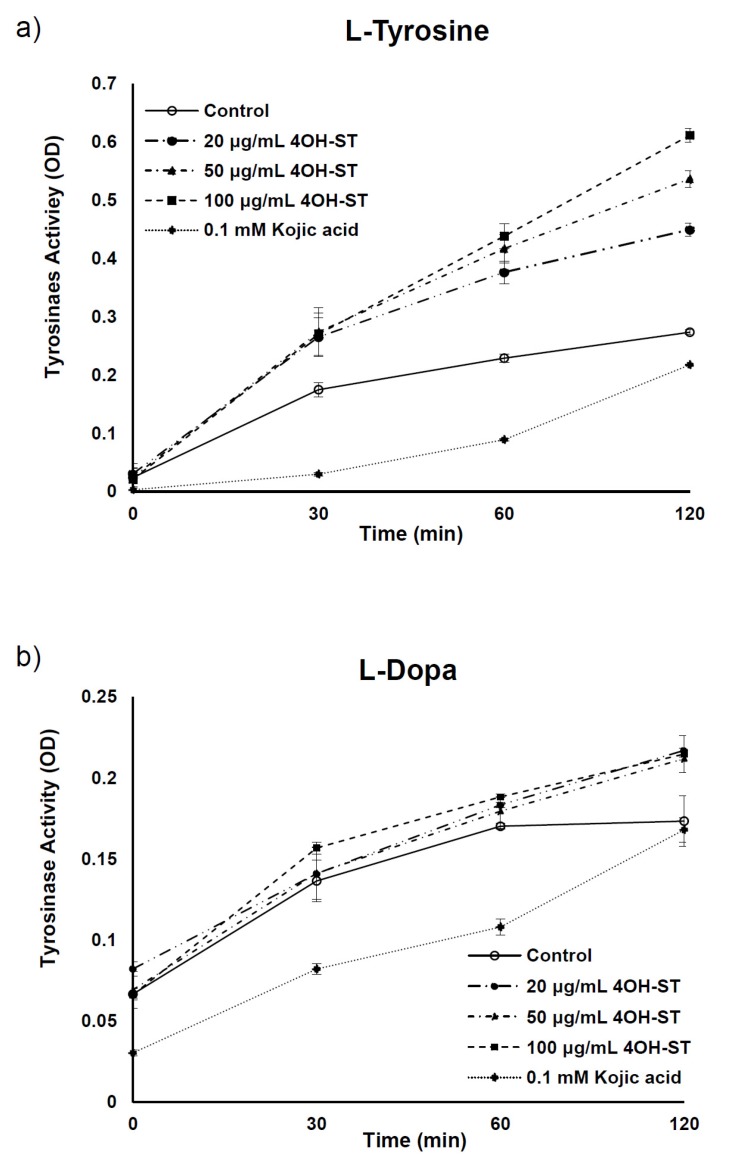
Effects of (-)-4-hydroxysattabacin (4OH-ST), **1**) on mushroom tyrosinase activity. Tyrosinase enzymatic activity was investigated using a mushroom tyrosinase assay with (**a**) l-tyrosine and (**b**) l-dopa (3,4-dihydroxyphenylalanine) as substrates. Data are presented as the mean ± SD (*n* = 3).

Generally, anti-melanogenic activities stem from the inhibition of melanogenic enzymes or suppression of their transcription [13,20]. To further examine the anti-pigmentary effect of (-)-4OH-ST (**1**), a cell-free mushroom tyrosinase assay was conducted to determine if enzymatic activities were affected. (-)-4OH-ST (**1**) did not affect the enzymatic activity of tyrosinase, while the positive control, kojic acid, inhibited its activity significantly (Figure 3a,b).

**Figure 4:**
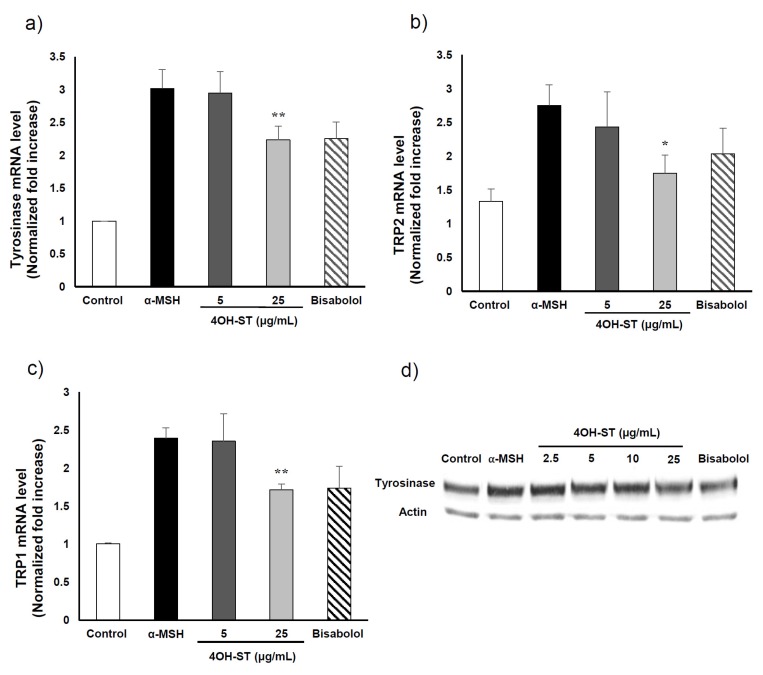
Effect of (-)-4-hydroxysattabacin (4OH-ST, **1**) on mRNA and protein level of B16F10 cells. mRNA expression levels of (**a**) tyrosinase; (**b**) TRP-2; and (**c**) TRP-1 in B16F10 cells were determined by real-time PCR; (**d**) Protein levels were determined by Western blotting. The cells were treated with bisabolol at 50 µM and (-)-4OH-ST (**1**) at the indicated concentrations for 24 h. Data are presented as the mean ± SD (*n* = 4, * *p* < 0.05 and ** *p* < 0.01).

We further tested whether (-)-4OH-ST (**1**) could regulate the expression of the melanogenic enzymes: tyrosinase, TRP-1, and TRP-2. The mRNA levels of these melanogenic enzymes were assessed by real-time PCR as indicated in the Experimental Section. Of note, (-)-4OH-ST (**1**) significantly attenuated the α-MSH-induced expression of tyrosinase, TRP-2, and TRP-1 (Figure 4A–C). Consistently, the protein level of tyrosinase was also suppressed by treatment with (-)-4OH-ST (**1**) (Figure 4d), suggesting that (-)-4OH-ST (**1**) might manifest hypopigmenting effects through suppression of the expression of melanogenic enzymes.

### 2.4. Effect of (-)-4-Hydroxysattabacin on the Melanin Synthesis and Cell Viability of Human Melanoma Cells

**Figure 5:**
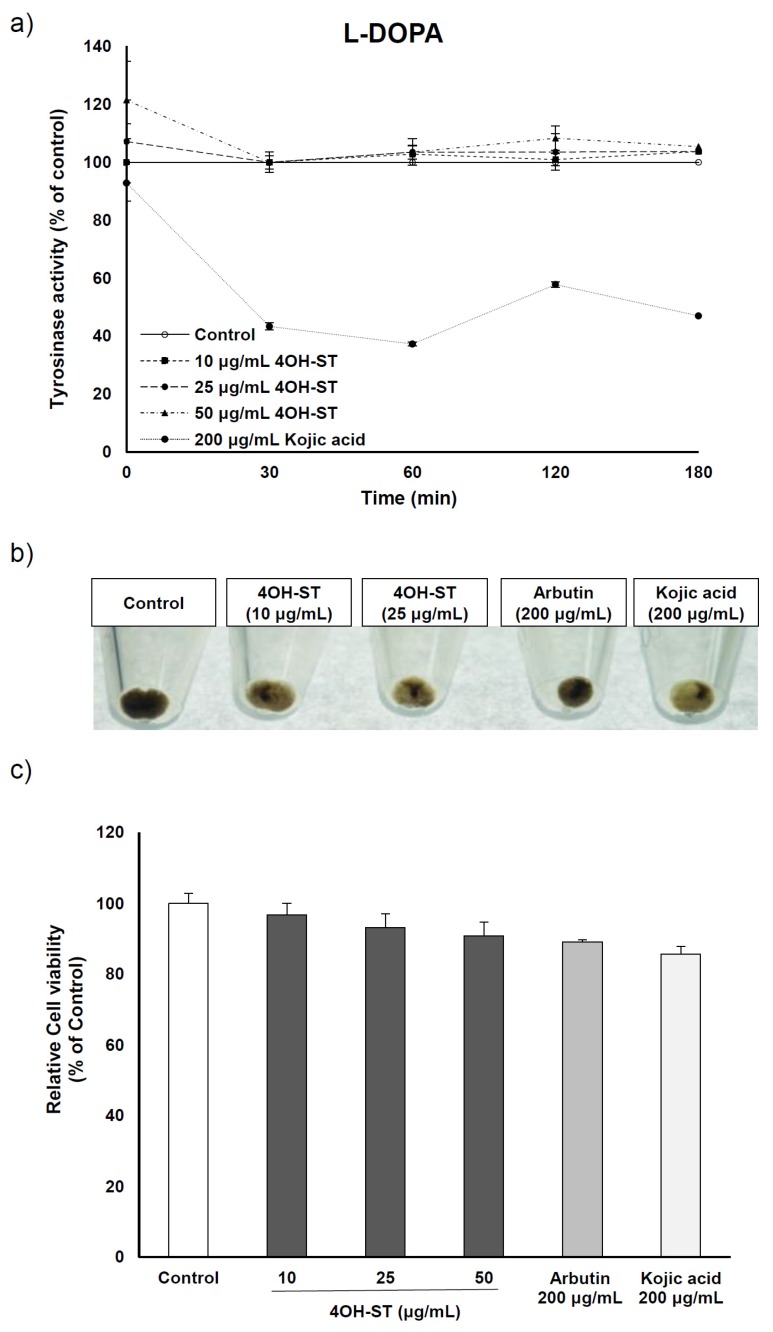
Effects of (-)-4-hydroxysattabacin (4OH-ST), **1**) treatment on MNT-1 cells. (**a**) Cellular tyrosinase activity data using l-dopa as substrate; (**b**) Macroscopic view of MNT-1 cell pellets; (**c**) Cell viability was determined by MTT assay. MNT-1 cells were stimulated by indicated materials with various concentrations for 72 h. Data are presented as the mean ± SD (*n* = 3).

The inhibitory effect of (-)-4OH-ST was further examined in human MNT-1 melanoma cells. Cellular tyrosinase activity assay was performed to examine the inhibitory effect on human tyrosinase activity using l-dopa as substrate. As expected, tyrosinase activity was unaffected by (-)-4OH-ST in comparison to the positive control, kojic acid (Figure 5a). However, when MNT-1 cells were treated with (-)-4OH-ST, melanin contents decreased in terms of pellet color (Figure 5b) with minimal effects on cell viability (Figure 5c), reflecting that 4OH-ST exhibits significant hypo-pigmenting effects in human melanocyte cell line, which were in line with the findings in murine melanoma cells.

### 2.5. Effects of (-)-4OH-ST Using an Artificial Human Epidermis, Keraskin^TM^ and MelanoDerm^TM^

**Figure 6:**
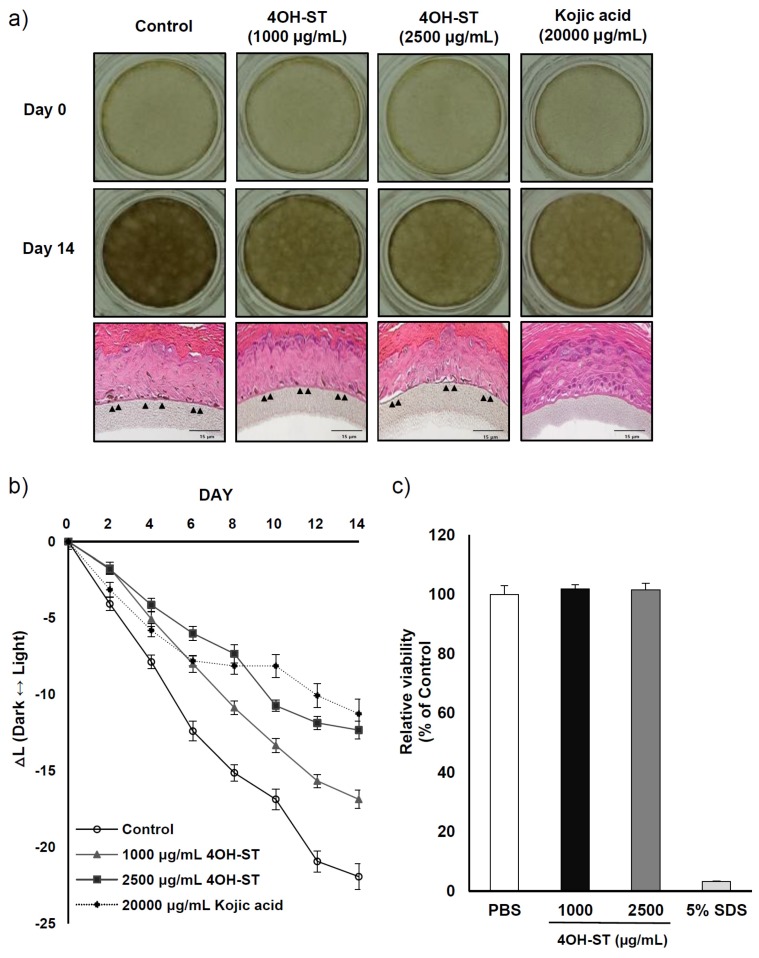
Effect of (-)-4-hydroxysattabacin (4OH-ST, **1**) on the Melanoderm^TM^ 3D skin model. (**a**) Color of 3D human skin tissue model (Melanoderm^TM^; MatTek) and hematoxylin and eosin (H & E) stained tissues; (**b**) The degree of hypopigmentation in Melanoderm^TM^ as measured by the ∆L value in skin tissue between baseline and 14 days after (-)-4OH-ST (**1**) or vehicle treatment; (**c**) Cell viability was further confirmed in a 3D reconstituted human epidermis model, Keraskin™, using WST-1 assay. Data are presented as the mean ± SD (*n* = 3).

Artificial human epidermis is widely used to examine the anti-pigmentary effects and safety of cosmetic ingredients in in vivo-like conditions. We examined whether (-)-4OH-ST (**1**) could attenuate melanin synthesis in an artificial pigmented skin model, Melanoderm^TM^ (MatTek, Ashland, MA, USA). Color alteration of the skin tissue was photographed every other day for 14 days, and the remaining tissues were stained to confirm melanin reduction. (-)-4OH-ST (**1**) lightened the colour of the skin tissue and significantly changed the degree of pigmentation, as represented by reduced ∆L (Figure 6a,b). Of note, the activities of 1000 μg/mL (-)-4OH-ST (**1**) were comparable to those of 20,000 μg/mL kojic acid, suggesting that (-)-4OH-ST (**1**) may be active in humans. The toxicity of (-)-4OH-ST was further investigated in a 3D reconstituted human epidermis model, Keraskin™ (Biosolution Co, Seoul, Korea) [21], which showed little effects on tissue viability (Figure 6c).

Hypopigmentation of the skin is important to alleviate hyperpigmentation disorders. Hyperpigmentation of skin is mainly caused by hyper-activated melanocytes that produce a larger than normal amount of melanin. In previous studies, natural products, such as arbutin, kojic acid, and aloesin [22,23] have shown to be effective against skin pigmentation [6]. Kojic acid, arbutin, and aloesin manifest hypopigmenting activities by inhibiting the enzymatic activity of tyrosinase. Kojic acid is known to be a slow-binding inhibitor of the hydroxylase activity of tyrosinase, whereas arbutin and aloesin reduce substrate affinity for the dopa oxidase catalytic site of tyrosinase as well as hydroxylase activities [22]. However, because these conventional agents may cause adverse skin reactions including erythema, burning, or pruritus with long-term exposure, there is a need to explore safer and more effective hypopigmenting agents [23,24,25]. In contrast to the mode of action of these conventional hypopigmenting agents, (-)-4OH-ST (**1**) suppressed the expression of melanogenic enzymes without affecting enzymatic activities and with minimal cytotoxicity.

Once melanin synthesis is initiated in melanocytes, melanosome packages are transferred along the dendrites to neighboring keratinocytes. Melanosomes translocated into keratinocytes aggregate around the nucleus to protect genetic materials against UV or ROS and are eventually degraded or removed from the skin when skin corneocytes detach. Furthermore, alteration of melanosome transfer may be the basis for several skin diseases, such as hyperpigmentation or hypopigmentation [26]. We suspect that α-MSH exposure leads to the formation of dendrites because α-MSH induces the synthesis of melanin in melanocytes. In this study, we verified morphological changes of melanocytes. As expected, α-MSH stimulated melanocytes showed extended dendrites in comparison to the control. Indeed, dendrite formation of melanocytes was prevented from (-)-4OH-ST (**1**) application.

A 3D human skin tissue model is widely used as an alternative to animal testing in order to investigate the whitening effect of chemical compounds [15,22], because the artificial structure of Melanoderm^TM^ mimics the components of human epidermal tissue [22], rendering animal tests unnecessary. Here, we confirmed the efficacy of (-)-4OH-ST (**1**) in a 3D skin model, further demonstrating the potent anti-melanogenic activities of (**1**). Furthermore, histological data from hematoxylin and eosin staining suggest that melanocytes shrank dramatically in treated tissue compared to untreated tissue (arrows in Figure 6a), which may be translated into potent whitening effects in human subjects.

## 3. Experimental Section

### 3.1. General Experimental Procedures

The optical rotation was measured using an Autopol III (Rudolph Research, Hackettstown, NJ, USA) polarimeter with a 5 cm cell. Circular dichroism (CD) spectra were collected in an Applied Photophysics Chirascan plus CD spectrometer with a 0.5 mm path-length rectangular cuvette. NMR spectra were recorded on a Varian Inova NMR spectrometer (500 and 125 MHz for ^1^H and ^13^C NMR, respectively), using the signals of the residual solvent protons and the solvent carbons as internal references (δ_H_ 7.24 and δ_C_ 77.0 ppm for CDCl_3_; δ_H_ 3.31 and δ_C_ 49.1 ppm for methanol-*d*_4_). Electron ionization mass spectrometry (EI-MS) spectra were measured on a JEOL (Akishima city, Japan), JMS-AX505WA mass spectrometer. Low-resolution liquid chromatography (LC)-MS data were measured using an Agilent Technologies 6120 quadrupole LC-MS system with a reversed phase column (Phenomenex Luna C18(2) 100 Å, 50 mm × 4.6 mm, 5 μm) at a flow rate of 1.0 mL/min. Column chromatographic separation was performed using Silica gel 60 (0.040~0.063 mm, Merch) under normal-phase methods. The fractions were purified using a WATERS^TM^ (Milford, MA, USA) 616 quaternary HPLC (high-performance liquid chromatography) pump, WATERS^TM^ 996 photodiode array detector using an Phenomenex Luna C18(2) (250 mm × 10 mm, 5 μm) reversed-phase HPLC column. α-MSH (α-Melanin Stimulating Hormone), arbutin, bisabolol, and DMSO were purchased from Sigma-Aldrich (St Louis, MO, USA). Cell culture medium and agents were from Thermo Scientific (Waltham, MA, USA). Primary antibodies for tyrosinase, and anti-rabbit secondary antibody HRP (horseradish peroxidase) conjugate were procured from Cell Signaling Technology (Beverly, MA, USA). Primary antibodies for β-actin were obtained from Abcam (Cambridge, UK). Anti-mouse secondary antibody HRP conjugate was obtained from KPL (Gaithersburg, MD, USA).

### 3.2. Strain and Cultivation

Strain SCO-147 (*Bacillus* sp.) was isolated from marine sediments collected off Suncheon Bay in Korea. The strain was classified by 16S rRNA analysis, which illustrated 99.8% identity to *Bacillus gibsonii*. Strain SCO-147 was cultured in 20 2.5-L Ultra Yield flasks each containing 1 L of the culture medium (10 g/L of soluble starch, 2 g/L of yeast, 4 g/L of peptone, 1.0 L of natural sea water) at 25 °C with shaking at 150 rpm. After 7 days, the broth was extracted with ethyl acetate (EtOAc) and evaporated under reduced pressure to yield the organic extract (1.5 g).

### 3.3. Extraction and Isolation

The crude extract (1.5 g) was subjected on open column chromatography on silica gel (30 g), eluted with a step gradient of dichloromethane and methanol. The dichloromethane/methanol (10:1) fraction was further fractionated by reversed-phase HPLC (Phenomenex Luna C-18 (2), 250 × 100 mm, 2.5 mL/min, 5 μm, 100 Å, UV = 210 nm) using an isocratic solvent system of 55% acetonitrile (CH_3_CN) in H_2_O to afford (-)-4-hydroxysattabacin (4OH-ST, **1**, 150.0 mg) and (-)-sattabacin (**2**, 100.0 mg).

4-Hydroxysattabacin (**1**): [α]_D_ = −29° (C 0.5, CHCl_3_), −14° (C 0.04, MeOH); ^1^H NMR (CDCl_3_, 500 MHz): δ_H_ 7.09 (2H, d, *J* = 8.1 Hz, H-2, H-6), 6.70 (2H, d, *J* = 8.1 Hz, H-3, H-5), 4.37 (1H, dd, *J* = 7.5, 4.4 Hz, H-2’), 3.08 (1H, dd, *J* = 14.3, 4.4 Hz, H-1’a), 2.76 (1H, dd, *J* = 14.3, 7.5 Hz, H-1’b), 2.38 (2H, d, *J* = 6.7, H-4’), 2.19 (1H, m, H-5’), 0.94 (6H, d, *J* = 6.7 Hz, Me-6’, Me-7’); ^13^C NMR (125 MHz, CDCl_3_): δ_C_ 211.6 (qC, C-3’), 154.9 (qC, C-4), 130.6 (CH, C-2, C-6), 128.5 (qC, C-1), 115.6 (CH, C-3, C-5), 77.8 (CH, C-2’), 47.6 (CH_2_, C-4’), 39.3 (CH_2_, C-1’), 24.9 (CH, C-5’), 22.9 (CH_3_, C-6’, C-7’); LC-MS *m*/*z*: 222 [M]^+^, 245 [M + Na]^+^.

Sattabacin (**2**): [α]_D_ = −50° (C 0.16, CHCl_3_); ^1^H NMR (500 MHz, MeOD): δ_H_ 7.32-7.20 (5H, m, H1–H4), 4.28 (1H, dd, *J* = 8.1, 4.8 Hz , H-2’), 3.07 (1H, dd, *J* = 13.8, 4.8 Hz, H-1’a), 2.80 (1H, dd, *J* = 13.8, 7.8 Hz, H-1’b), 2.42 (2H, dd, *J* = 6.9, 2.1 Hz, H-4’), 2.11 (1H, m, H-5’), 0.91 (6H, d, *J* = 6.6 Hz, H-6’, H-7’); ^13^C NMR (125 MHz, MeOD): δ_C_ 214.4 (qC, C-3’), 139.2 (qC, C-1), 130.8 (CH, C-2, C-6), 129.6 (CH, C-3, C-5), 127.8 (CH, C-4), 79.4 (CH, C-2’), 49.3 (CH2, C-4’), 41.2 (CH2, C-1’), 25.4 (CH, C-5’), 23.3 (CH3, C-6’), 23.2 (CH3, C-7’); LR-MS *m*/*z*: 207 [M + H]^+^, 229 [M + Na]^+^.

### 3.4. Cell Culture

The B16F10 cell line from C57BL/6 mice was purchased from ATCC (Manassas, VA, USA). Cells were maintained in standard culture conditions, Dulbecco’s Modified Eagle’s Medium (DMEM) supplemented with antibiotics (100 U/mL of penicillin A and 100 U/mL of streptomycin) and 10% fetal bovine serum (FBS) at 37 °C in a humidified atmosphere containing 5% CO_2_. At 80% cell confluence, adherent cells were detached with a solution of trypsin (Hyclone, South Logan, UT, USA).

MNT-1 cells were maintained in minimum essential medium supplemented with 10% DMEM, 20% fetal bovine serum (FBS), 1 M HEPES, and streptomycin-penicillin (100 U/mL each) at 37 °C in a humidified atmosphere containing 5% CO_2_. Monolayers of 80% confluence cells were cultured with 0.05% trypsin (Hyclone, South Logan, UT, USA).

### 3.5. Melanin Assay and Cell Viability Assay (WST-1 or MTT)

One day prior to an experiment, B16F10 cells were seeded at 2 × 10^4^ cells/well into 48-well plates. The serum-starved cells were treated with various concentrations of (-)-4OH-ST (**1**) in culture medium containing 0.5% dimethyl sulfoxide (DMSO) and 200 nM α-MSH for 72 h. Here, α-MSH was used for inducing melanin synthesis in all experiments. α-MSH untreated cells were used as a negative control and arbutin treated cells were used as a positive control. After cells were dissolved in 200 µL of 1*N* NaOH at 60 °C for 1 h in the dark, the total melanin content was measured by absorbance at 405 nm using a microplate reader (Spectra max 190, Molecular Devices, Sunnyvale, CA, USA). WST-1 (4-[3-(indophenyl)-2-(4-nitrophenyl)-2H-5-tetrazolio]-1,3-benzene disulfonate) (Roche, Indianapolis, IN, USA) solution or 3-(4,5-dimethylthiazol-2-yl)-2,5-diphenyltetrazolium bromide (MTT) was used to investigate cell viability. 4OH-ST (**1**) treated B16F10 cells were incubated with 200 µL of WST-1 solution for 2.5 h at 37 °C. 5% CO_2_ in the dark. 100 µL of supernatant was transferred into each well of 96 well plate, and absorbance was measured at 450 nm. All measurements were performed in triplicate. To assay mitochondrial reduction of MTT, B16F10 or MNT-1 cells were incubated with 0.25 mL of 0.5 mg/mL MTT solution in DMEM for 2 h at 37 °C. The blue formazan dye was dissolved in 300 L of DMSO for 30 min and 200 L of supernatant was measured as the absorption value at 540 nm.

### 3.6. Mushroom Tyrosinase Inhibition Assay

In vitro cell-free assay system was performed to determine whether 4-hydroxysattabacin showed any direct inhibitory effect against tyrosinase, a key enzyme in melanogenesis. 180 µL of 0.03% tyrosine in 0.1 M potassium phosphate was added to 20 µL of mushroom tyrosinase (250 units) and 180 µL of 0.2% L-dopa in 0.1 M potassium phosphate was mixed with 50 units of mushroom tyrosinase. 2 µL of DMSO (final 0.5%) containing compound was added and incubated at 37 °C for indicated time. The absorbance was measured at 475 nm (Spectra max 190, Molecular Devices, Sunnyvale, CA, USA).

### 3.7. RNA Isolation

B16F10 cells were washed with phosphate-buffered saline after 12 h exposure of (-)-4-OH ST (**1**), and were lysed using Trizol (Invitrogen, CA, USA). After the addition of chloroform, samples were centrifuged at 12,000 rpm for 10 min. The aqueous phase was mixed with isopropanol and RNA pellets were collected by centrifugation (12,000 rpm, 15 min, 4 °C). RNA pellets were washed with 70% ethanol and dissolved in RNase-free, DEPC (diethyl pyrocarbonate)-treated water (Waltham, MA, USA). The RNA yield was estimated by determining the optical density at 260 nm with a NanoDrop 1000 spectrophotometer (NanoDrop Technologies, INC., Wilmington, DE, USA).

### 3.8. Real-Time PCR

Relative expression levels of mRNAs were measured by quantitative real-time PCR. cDNA was synthesized from 1250 ng of total RNA with oligo(dT) (Bioelpis, Seoul, Korea). SYBR Green PCR master mix and a StepOnePlus^TM^ Real-time PCR machine (Applied Biosystems, Warrington, UK) were used in each reaction. The sequence of primers was as follows: forward tyrosinase, 5′-G GGCCCAAATTGTACAGAGA-3′; reverse tyrosinase, 5′-ATGGGTGTTGACCATTGTT-3′; forward TRP-1, 5′-GTTCAATGGCCAGGTCAGGA-3′; reverse TRP-1, 5′-CAGACAAGAAGCAACCCCGA-3′; forward TRP-2 5′-GCTTGGAGCAGCAAGACAAG-3′; reverse TRP-2 5′-ATTACACAGTGTGACCCGGC-3′. Cycling parameters were 50 °C for 2 min, 95 °C for 10 min, 40 cycles of 95 °C 15 s, and 50 °C 1 min.

### 3.9. Western Blot Analysis

After 24 h of exposure to 4OH-ST (**1**), B16F10 cells were homogenized in RIPA buffer (Sigma, MO, USA) containing 1% protease inhibitor cocktail and a phosphatase inhibitor cocktail. The supernatant of the homogenate was collected and the protein concentration was determined by BCA assay. Equal amount of proteins was subjected to 10% sodium dodecyl sulfate-polyacrylamide gel electrophoresis (SDS-PAGE) and transferred to nitrocellulose membranes (Amersham, Buckinghamshire, UK). After being blocked at room temperature in 2% skim milk for 1 h, the blots were probed with each of the following primary antibodies against each target protein in 1 × TBST for 2 h at room temperature: mouse anti-tyrosinase monoclonal antibody (1:2000 dilution: Abfrontier, Seoul, Korea). HRP-conjugated secondary antibodies (KPL, Gaithersburg, MD, USA) were used to detect bound antibodies, and the immune reactive bands were visualized using ECL Western blotting detection reagents (Amersham Biosciences, Little Chalfont, UK) and an X-ray film. β-actin (antibody, Santa Cruz Biotechnology, Santa Cruz, CA, USA) was used as a control for immunoblotting.

### 3.10. Hypopigmenting Effect of 4OH-ST (1) Using the Pigmented Human Epidermal 3D Skin Model, Melanoderm^TM^

Melanoderm^TM^ (MatTek) is an artificial human epidermis consisting of normal human derived epidermal keratinocytes and normal human derived melanocytes (NHM) that exhibit uniform and highly reproducible morphological and ultrastructural characteristics. Tissues containing NHM derived from black donors become increasingly pigmented with retention of normal epithelial morphology. Briefly, Melanoderm^TM^ was pre-incubated for 24 h, then treated with the indicated compounds every other day for 14 days. The ∆L value was analyzed with Adobe Photoshop CC 2015 software. (San Jose, CA, USA).

### 3.11. Statistics Analysis

Data are expressed as mean ± standard error of the mean of three or more independent experiments. The statistical significance of differences between groups was assessed using a two-sided Student’s t-test. *p* values < 0.05 were considered statistically significant.

## 4. Conclusions

We demonstrated that (-)-4OH-ST (**1**), a major compound from the extract of culture broth of *Bacillus* sp. can modulate melanogenesis through down-regulation of the expression of melanogenic enzymes with minimal cytotoxicity in the murine melanoma cell, B16F10, and human melanoma cell, MNT-1. In addition, the anti-pigmentary effect of (-)-4OH-ST (**1**) was confirmed in an artificial human skin model. Of note, the potency and efficacy of (-)-4OH-ST (**1**) were comparable or superior to those of arbutin, bisabolol and kojic acid, indicating that it could be a promising hypopigmenting agent.

## Supplementary Materials

The ^1^H, ^13^C NMR, CD spectral data of (-)-4-hydrosattabacin (4OH-ST, **1**) and (-)-sattabacin (**2**), and the biological activity of the crude extract of SCO-147 are available online at www.mdpi.com/1660-3397/15/5/138/s1.

## References

[1] Costin G.E., Hearing V.J. (2007). Human skin pigmentation: Melanocytes modulate skin color in response to stress. FASEB J..

[2] Slominski A., Tobin D.J., Shibahara S., Wortsman J. (2004). Melanin pigmentation in mammalian skin and its hormonal regulation. Physiol. Rev..

[3] Ito S., Wakamatsu K. (2003). Quantitative analysis of eumelanin and pheomelanin in humans, mice, and other animals: A comparative review. Pigment Cell Res..

[4] Briganti S., Camera E., Picardo M. (2003). Chemical and Instrumenatal Approaches to Treat Hyperpigmentation. Pigment Cell Res..

[5] Lee M., Park H., Jeon S.W., Bang J., Chung K.Y., Choi D.W., Kim E., Lim K.M. (2015). Novel anti-melanogenic hexapeptoids, PAL-10 and PAL-12. Arch. Dermatol. Res..

[6] Zhu W., Gao J. (2008). The use of botanical extracts as topical skin-lightening agents for the improvement of skin pigmentation disorders. J. Investig. Dermatol. Symp. Proc..

[7] Dessinioti C., Stratigos A.J., Rigopoulos D., Katsambas A.D. (2009). A review of genetic disorders of hypopigmentation: Lessons learned from the biology of melanocytes. Exp. Dermatol..

[8] Pillaiyar T., Manickam M., Jung S.H. (2017). Downregulation of melanogenesis: Drug discovery and therapeutic options. Drug Discov. Today.

[9] Pillaiyar T., Manickam M., Namasivayam V. (2017). Skin whitening agents: Medicinal chemistry perspective of tyrosinase inhibitors. J. Enzyme Inhib. Med. Chem..

[10] Gillbro J.M., Olsson M.J. (2011). The melanogenesis and mechanisms of skin-lightening agents-existing and new approaches. Int. J. Cosmet. Sci..

[11] Hedley S.J., Gawkrodger D.J., Weetman A.P., Macneil S. (1998). alpha-MSH and melanogenesis in normal human adult melanocytes. Pigment Cell Res..

[12] Siegrist W., Solca F., Stutz S., Giuffre L., Carrel S., Girard J., Eberle A.N. (1989). Characterization of receptors for a-Melanocyte-stimulating Hormone on Human Melanoma Cells. Cancer Res..

[13] Nishioka E., Funasaka Y., Kondoh H., Chakraborty A.K., Mishima Y., Ichihashi M. (1999). Expression of tyrosinase, TRP-1 and TRP-2 in ultraviolet-irradiated human melanomas and melanocytes: TRP-2 protects melanoma cells from ultraviolet B induced apoptosis. Melanoma Res..

[14] Aoki Y., Tanigawa T., Abe H., Fujiwara Y. (2007). Melanogenesis inhibition by an oolong tea extract in b16 mouse melanoma cells and UV-induced skin pigmentation in brownish guinea pigs. Biosci. Biotechnol. Biochem..

[15] Yoon T.J., Lei T.C., Yamaguchi Y., Batzer J., Wolber R., Hearing V.J. (2003). Reconstituted 3-dimensional human skin of various ethnic origins as an in vitro model for studies of pigmentation. Anal. Biochem..

[16] Lampis G., Deidda D., Maullu C., Madeddu M.A., Pompei R. (1995). Sattabacins and Sattazolins: New Biologically Active Compounds with Antiviral Properties Extracted from a *Bacillus* sp.. J. Antibiot..

[17] Mancha S.R., Regnery C.M., Dahlke J.R., Miller K.A., Blake D.J. (2013). Antiviral activity of (+)-sattabacin against varicella zoster. Bioorg. Med. Chem. Lett..

[18] Xuemei L., Yu T.-K., Kwak J., Son B.-Y., Seo Y., Zee O.-P., Ahn J.-W. (2008). Soraphinol C, a New Free -Radical Scavenger from *Sorangium cellulosum*. J. Microbiol. Biotechnol..

[19] Park J-S., Kagaya N., Hashimoto J., Izumikawa M., Yabe S., Shin-ya K., Nishiyama M., Kuzuyama T. (2014). Identification and Biosynthesis of New Acyloins from Bacterium *Thermosporothrix hazakensis* SK20-1^T^. Chembiochem.

[20] Kim S.S., Kim M.J., Choi Y.H., Kim B.K., Kim K.S., Park K.J., Park S.M., Lee N.H., Hyun C.G. (2013). Down-regulation of tyrosinase, TRP-1, TRP-2 and MITF expressions by citrus press-cakes in murine B16 F10 melanoma. Asian Pac. J. Trop. Biomed..

[21] Jung K.-M., Lee S.-H., Jang W.-H., Jung H.-S., Heo Y., Park Y.-H., Bae S., Lim K.-M., Seo S. (2014). Keraskin-VM: A novel reconstructed human epidermis model for skin irritation tests. Toxicol. In Vitro.

[22] Jones K., Hughes J., Hong M., Jia Q., Orndorff S. (2002). Modulation of melanogenesis by aloesin: A competitive inhibitor of tyrosinase. Pigment Cell Res..

[23] Kim M., Shin S., Lee J.-A., Park D., Lee J., Jung E (2015). Inhibition of melanogenesis by *Gaillardia aristata* flower extract. BMC Complement. Altern. Med..

[24] Nakagawa M., Kawai K., Kawai K. (1995). Contact allergy to kojic acid in skin care products. Contact Dermat..

[25] Garcia-Gavin J., Gonzalez-Vilas D., Fernandez-Redondo V., Toribio J. (2010). Pigmented contact dermatitis due to kojic acid. A paradoxical side effect of a skin lightener. Contact Dermat..

[26] Boissy R.E. (2003). Melanosome transfer to and translocation in the keratinocyte. Exp. Dermatol..

