# Supplementary material for: Anti-Pigmentary Effect of (-)-4-Hydroxysattabacin from the Marine-Derived Bacterium Bacillus sp
Source: Mar Drugs. 2017 May 13;15(5):138. doi: 10.3390/md15050138 (PMC5450544; doi:10.3390/md15050138)

Supplementary Materials: Anti-pigmentary Effect of (−)-4-Hydroxysattabacin from a Marine-derived Bacterium *Bacillus* sp.

Kyuri Kim,[†] *Alain S. Leutou,*[‡] *Haein Jeong,*[‡] *Dayoung Kim,*[‡] Chi *Nam Seong,*[⊥] *Sang-Jip Nam,**[,‡] *and Kyung-Min Lim**[,†]

[†]College of Pharmacy, Ewha Womans University, Seoul, 120-750, Republic of Korea

[‡]Department of Chemistry and Nano Science, Global Top 5 program, Ewha Womans University, Seoul 03760, Korea

[⊥]Department of Biology, College of Life Science and Natural Resource, Sunchon National University, Suncheon, 540-742, Republic of Korea

Table of Contents

Figure S1. Effect of crude extract of *Bacillus* sp., SC0147, on melanin contents and cell viability of B16F10 cell …………………………………………………………………………………… S3

Figure S2. ^{1}H NMR Spectrum (500 MHz) of 4-Hydroxysattabacin in $CDCl_3$.................................. S4

Figure S3. ^{13}C NMR Spectrum (125 MHz) of 4-Hydroxysattabacin in $CDCl_3$................................ S5

Figure S4. ^{1}H NMR Spectrum (500 MHz) of Sattabacin in MeOD... S6

Figure S5. ^{13}C NMR Spectrum (125 MHz) of Sattabacin in MeOD.. S7

Figure S6. CD Spectra of 4-Hydroxysattabacin and Sattabacin………………………………………. S8

Figure S1. Effect of crude extract of *Bacillus* sp., SC0147, on melanin contents and cell viability of B16F10 cell

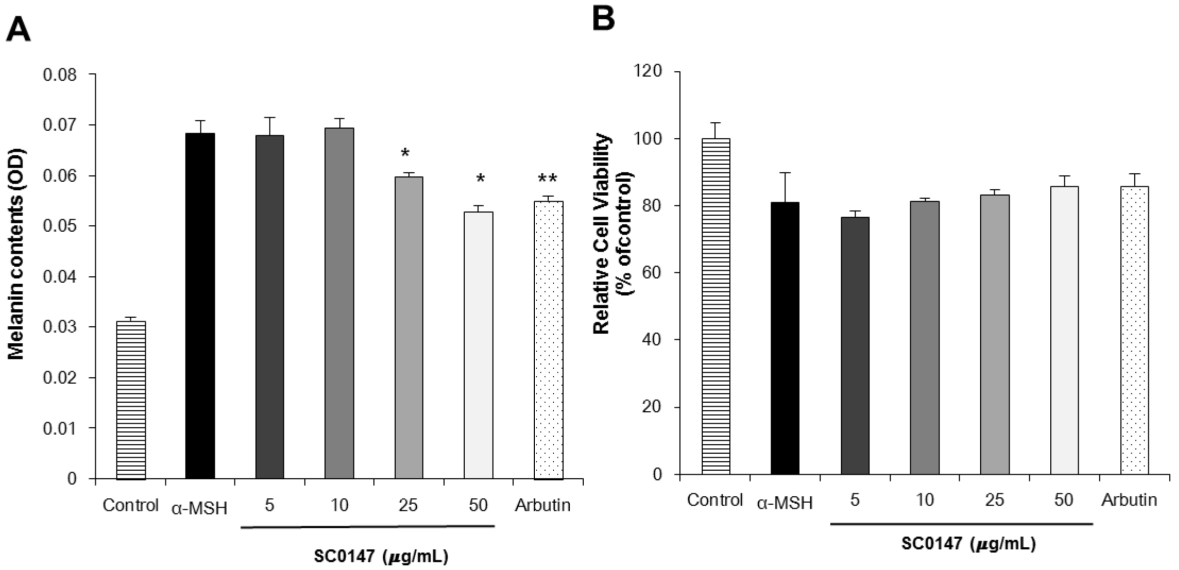

(A) Measure of melanin contents (N=3) by 1M NaOH, and (B) measure of Cell viability by WST-1 assay. B16 cells were treated with 0.5 μM α-MSH in the presence or absence of indicated concentration of SC0147 for 72 h.

Figure S2. ^{1}H NMR Spectrum (500 MHz) of 4-Hydroxysattabacin in $CDCl_3$

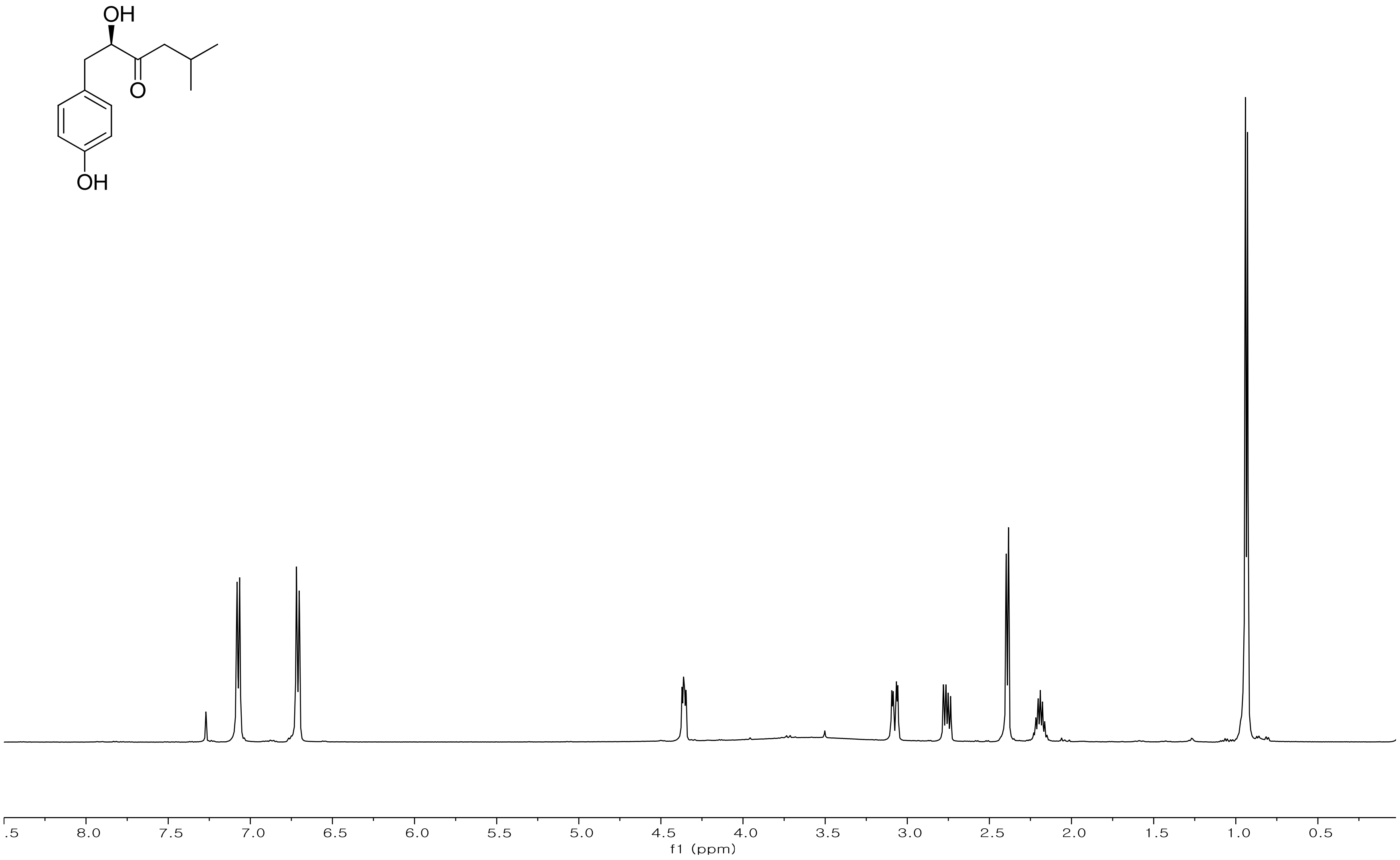

Figure S3. ^{13}C NMR Spectrum (125 MHz) of 4-Hydroxysattabacin in $CDCl_3$

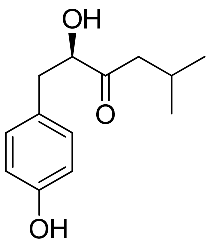

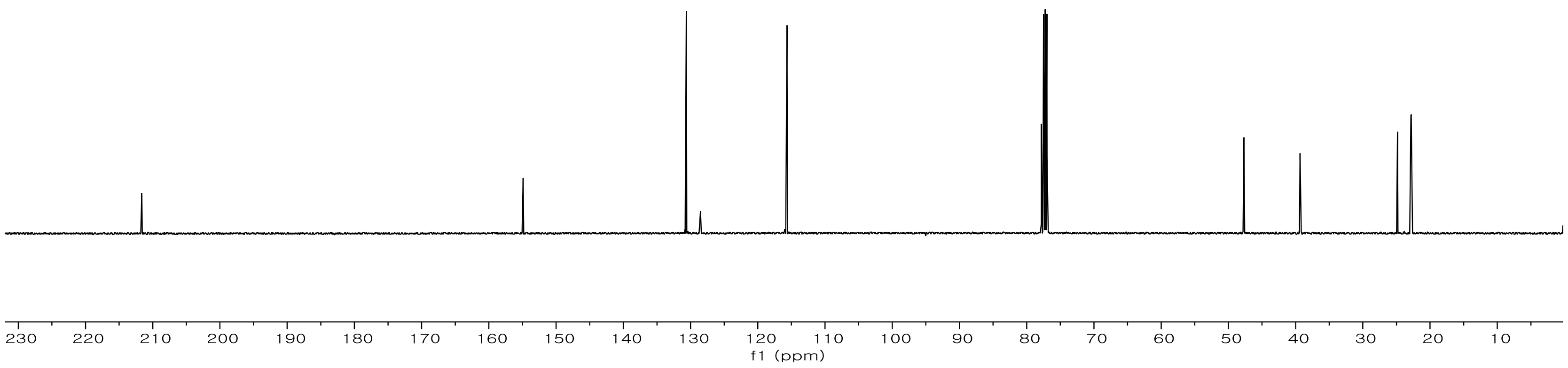

Figure S4. ^{1}H NMR Spectrum (500 MHz) of Sattabacin in MeOD

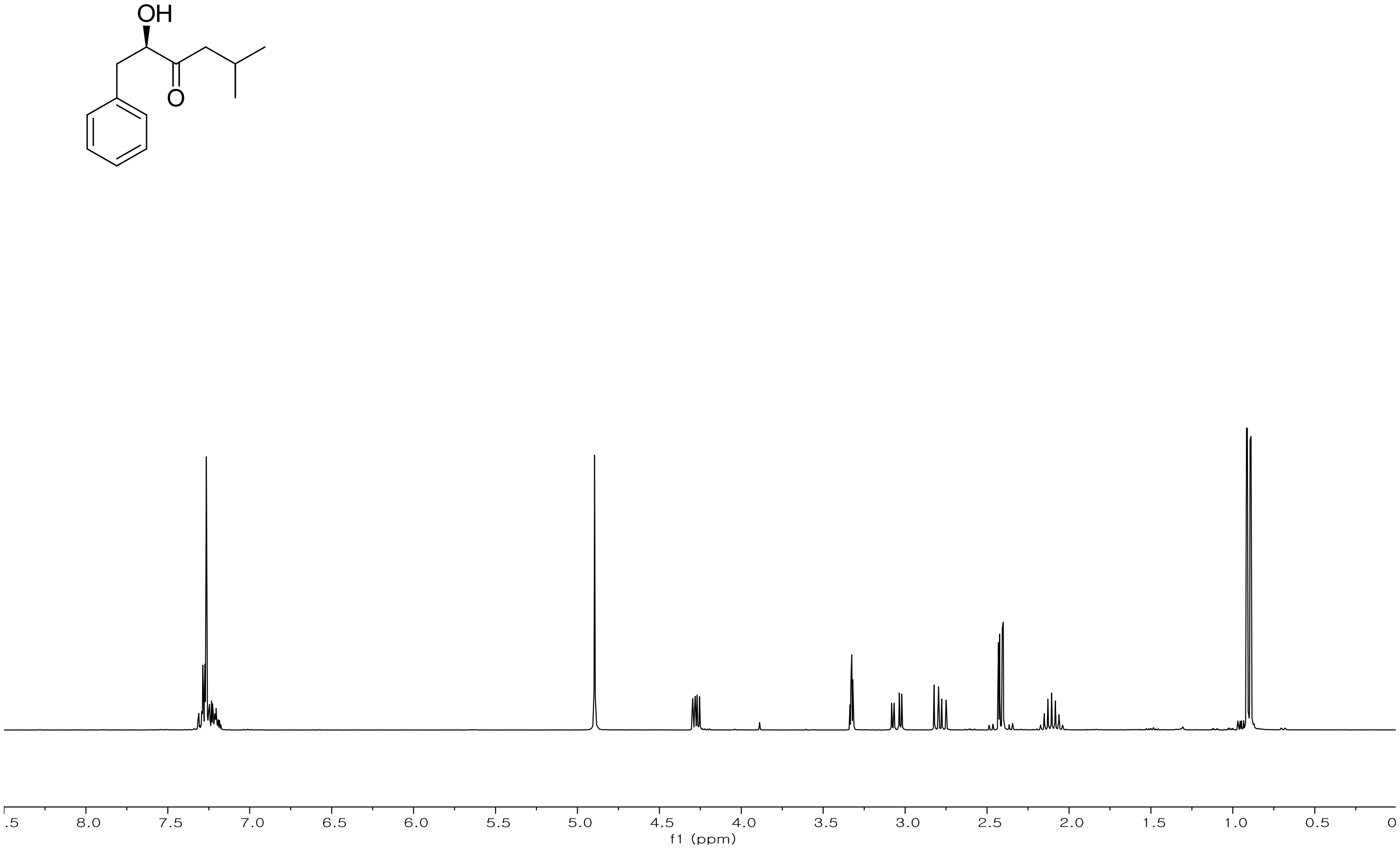

Figure S5. ^{13}C NMR Spectrum (125 MHz) of Sattabacin in MeOD

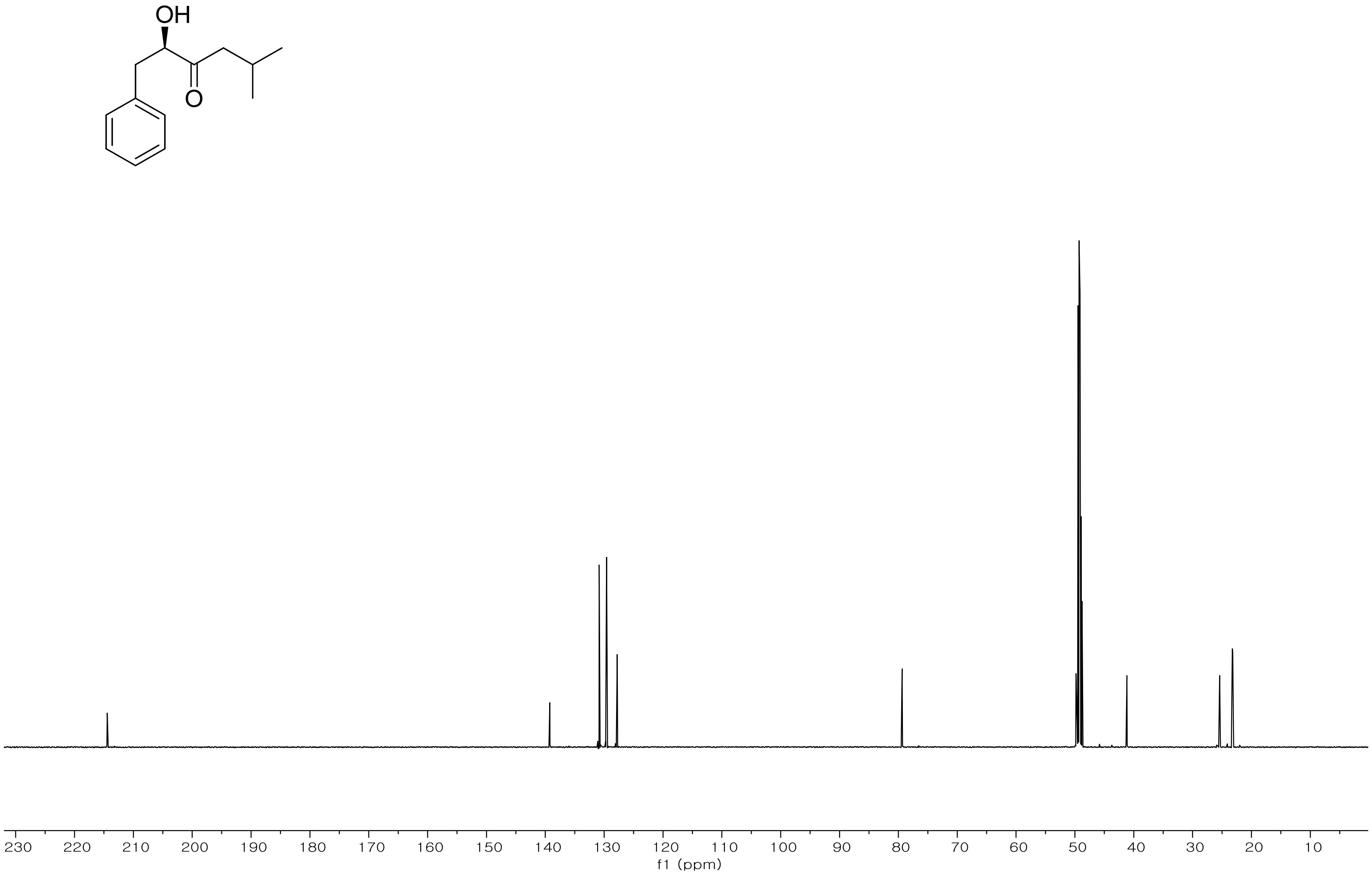

Figure S6. CD Spectra of 4-Hydroxysattabacin and Sattabacin

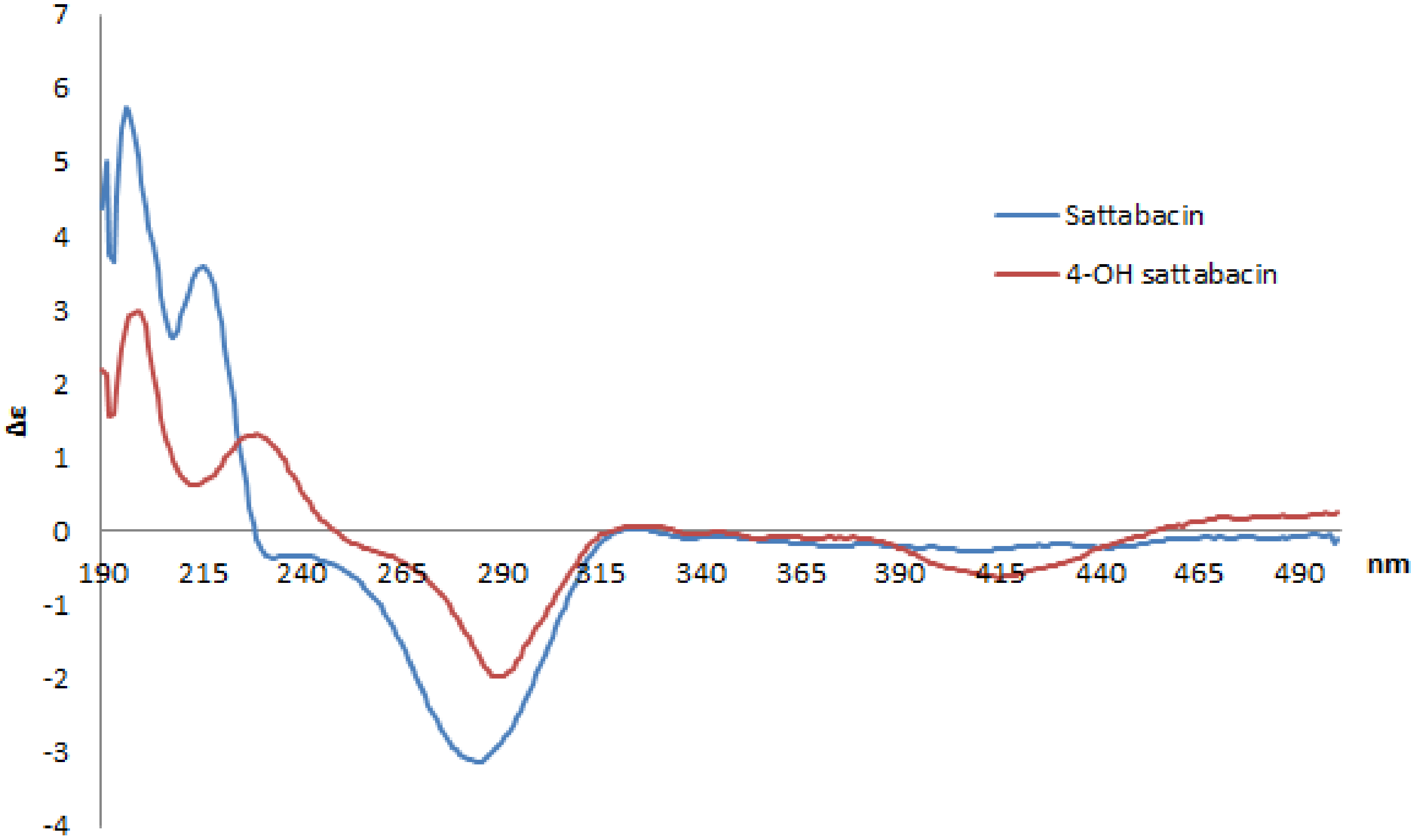

Supplement: Supplementary file 1 [file marinedrugs-15-00138-s001.pdf]
